# Uncovering the antifungal activities of wild apple-associated bacteria against two canker-causing fungi, *Cytospora mali* and *C. parasitica*

**DOI:** 10.1038/s41598-024-56969-4

**Published:** 2024-03-15

**Authors:** Tohir A. Bozorov, Zokir O. Toshmatov, Gulnaz Kahar, Surayya M. Muhammad, Xiaojie Liu, Daoyuan Zhang, Ilkham S. Aytenov, Khurshid S. Turakulov

**Affiliations:** 1 State Key Laboratory of Desert and Oasis Ecology, Key Laboratory of Ecological Safety and Sustainable Development in Arid Lands, Xinjiang Institute of Ecology and Geography, Chinese Academy of Sciences, Ürümqi, China; 2 Xinjiang Key Lab of Conservation and Utilization of Plant Gene Resources, Xinjiang Institute of Ecology and Geography, Chinese Academy of Sciences, Ürümqi, 830011 China; 3 Turpan Eremophytes Botanical Garden, Chinese Academy of Sciences, Turpan, 838008 China; 4 Laboratory of Molecular and Biochemical Genetics, Institute of Genetics and Plants Experimental Biology, Academy of Sciences of the Republic of Uzbekistan, Tashkent, Uzbekistan

**Keywords:** Antifungal compound, Apple disease, Pathogenic fungi, Phenazine, *Pseudomonas synxantha*, Environmental microbiology, Antifungal agents

## Abstract

*Cytospora* canker has become a devastating disease of apple species worldwide, and in severe cases, it may cause dieback of entire trees. The aim of this study was to characterize the diversity of cultivable bacteria from the wild apple microbiota and to determine their antifungal ability against the canker-causing pathogenic fungi *Cytospora mali* and *C. parasitica*. Five bacterial strains belonging to the species *Bacillus amyloliquefaciens, B. atrophaeus, B. methylotrophicus, B. mojavensis,* and *Pseudomonas synxantha* showed strong antagonistic effects against pathogenic fungi. Therefore, since the abovementioned *Bacillus* species produce known antifungal compounds, we characterized the antifungal compounds produced by *Ps. synxantha*. Bacteria grown on nutritional liquid medium were dehydrated, and the active compound from the crude extract was isolated and analysed via a range of chromatographic processes. High-performance liquid chromatography, mass spectrometry, and nuclear magnetic resonance analyses revealed a bioactive antifungal compound, phenazine-1-carboxylic acid (PCA). The minimum inhibitory concentration (MIC) demonstrated that PCA inhibited mycelial growth, with a MIC of 10 mg mL^−1^. The results suggested that PCA could be used as a potential compound to control *C. mali* and *C. malicola,* and it is a potential alternative for postharvest control of canker disease.

## Introduction

Apple, a nutritionally important fruit, is one of the most widely planted and consumed fruits worldwide^1,2^. China is considered the top apple-producing country in the world, with total production exceeding 45 million tons in 2023, according to the USDA (https://apps.fas.usda.gov/psdonline/circulars/fruit.pdf). The wild apple, *Malus sieversii* (Ledeb.) Roem. is an endemic species native to Central Asia (including western China)^3^. Moreover, *M. sieversii* is believed to be the primary progenitor of cultivated apples^4^. It is regarded as an internationally important genetic resource for apple breeding due to its extensive genetic diversity.

Plant pathogens, including viruses, bacteria, fungi, and nematodes, are responsible for substantial crop losses and damage to plants worldwide^5,6^. Fungal disease, on the other hand, poses a serious threat to domestic apples, particularly *M. sieversii,* and their wild relatives^7,8^. Up to 50% of the annual apple harvest is lost each year because of biotic and abiotic stressors. In the 1990s, an apple wood-borer, *Agrilus mali,* heavily attacked the wild apple population in the Tianshan Forest (Western China)^9,10^; since then, 40% of the forest area has been damaged^11^. Therefore, it is important to develop in situ and ex situ conservation methods for wild apple species.

Apple dieback is caused by different canker fungal species and is one of the most destructive diseases of *Malus* species worldwide^12–17^. Most of the fungal species of the Botryosphaeriaceae and Valsaceae families have been isolated from apple trees exhibiting canker symptoms^17–20^. Canker pathogens infect their host through wounds and colonize vascular tissue, where they have a negative effect on the vascular system, causing the trunk, twigs, and branches to eventually die^21^. Among canker-causative fungal pathogens, *Cytospora mali* is considered one of the most important threats to wild apple forests^16,18,22^. Unfortunately, there are no known fungicides for Cytospora canker, but effective management of infection and/or spread of the disease involves pruning canker-damaged wood from orchards during months with low relative humidity and low temperature averages^23,24^.

The development of biological control programs is important in forestry and agriculture, especially for the exploration of biologically active natural products with high biological and economic potential^25,26^. Various methods and strategies are used for plant disease management, such as physical (crop rotation, sanitation, quarantining, mulching, pruning, trapping and barrier, heat and cold treatments, UV irradiation and others), chemical (fungicides, chemical elicitors that induce resistance, and plant growth regulators that enhance plant immunity), and biological (antagonistic organisms, breeding and creation of resistant plants) methods^27–30^. These strategies have remarkably improved crop productivity at various disease incidence levels. Since gaining knowledge on the negative impact of chemicals on the environment, the world has started to reduce the use of agrochemicals in pest management and to seek eco-friendly and alternative control methods. Alternative disease control methods include nontraditional methods, i.e., alternatives to synthetic chemical pesticides based on the application of beneficial organisms known as biological control agents^31^. Biological control is based on the identification of naturally antagonistic organisms, such as natural enemies of insects or antagonists of pathogens (bacteria, fungi), which come from the same area of origin or are climatically close to an area where pathogens or pests occur. This provides ecologically favourable conditions for the existence of biological antagonists^32–34^. There are limitations to the colonization or competition of selected biological control agents due to their origin in different environments^35^.

Investigating host-associated bacteria is crucial for the development of a biological control program. Using microbiological and analytical techniques, the aim of this study was to investigate the antifungal activity of bacteria associated with wild apples against the canker-causing fungal pathogens *C. mali* and *C. parasitica*. Among the majority of identified bacterial species, only a few *Bacillus* species showed strong antagonistic effects on pathogenic fungi. Phenazine-1-carboxylic acid (PCA) produced by host-associated *Ps. synxantha* showed strong antifungal activity at the lowest minimum inhibitory concentration (MIC). This bacterium, particularly the well-characterized *Bacillus* species, shows potential as a biological control agent. The bioactive compounds or bacterial extracts derived from this bacterium could be employed as fungicides for controlling canker disease.

## Results

### Identification of cultivable wild apple-associated bacteria

**Figure 1:**
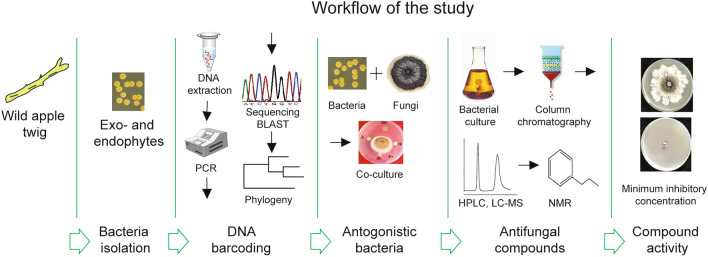
A graphical abstract depicting the determination of bacteria-antagonists of pathogenic fungi.

To determine the antagonistic effects of apple bacteria on pathogenic *C. mali* and *C. parasitica*, we applied microbiological, molecular, and analytical tools. For this purpose, we developed an exploratory strategy for stepwise identification of wild apple twig-associated bacteria and screening their antagonistic ability against pathogenic fungi, as depicted in the graphical abstract (Fig. 1).

**Figure 2:**
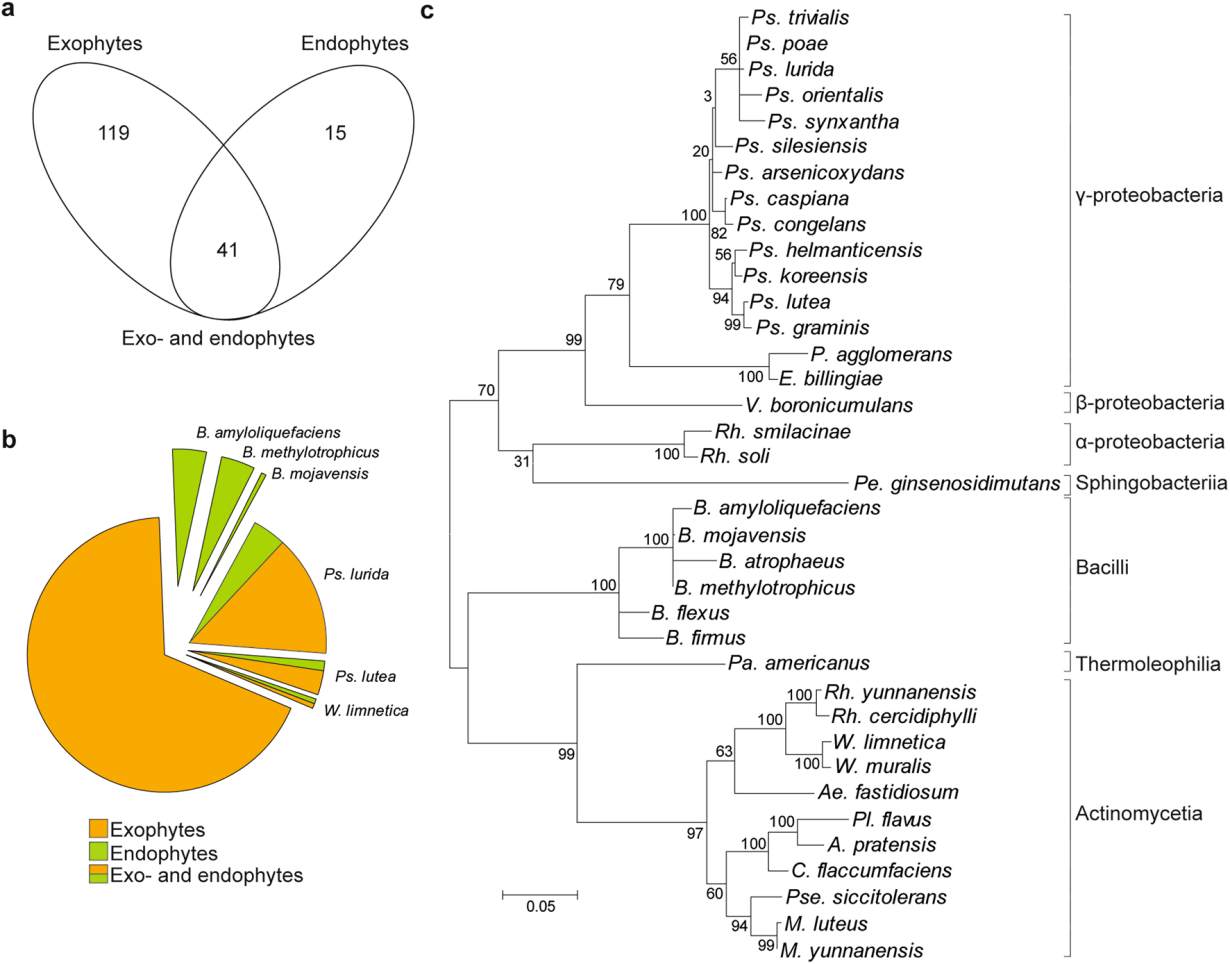
Diversity, abundance, and clustering analysis of cultivable wild apple twig associated bacteria. The Venn diagram indicates the distribution of cultivable bacterial isolates among exo- and endophytes (**a**). The pie chart depicts the distribution of bacterial isolates. Bacterial species in the yellow and green pie pieces indicate the respective exophytic and endophytic bacterial isolates. The pie chart with a mix of green and orange shows bacterial isolates at the species level that were found both on the surface and inside of twigs (**b**). In the clustering analysis of wild apple-associated bacteria, the maximum likelihood was inferred using the neighbour-joining method. The percentage of replicate trees in which the associated taxa clustered together in the bootstrap test (5000 replicates) is shown next to the branches. The evolutionary distances were computed using the Tajima–Nei method and are in units of the number of base substitutions per site. The distance scale represents the number of differences between the sequences. Evolutionary analyses were conducted in MEGA 7 (**c**).

Bacteria from wild apple twigs were isolated to identify bacterial antagonists of pathogenic fungi. A total of 175 microbial isolates were obtained, and bacterial colonies were randomly selected based on their morphological features. Fifteen of the isolates were endophytic bacteria, while the remaining 119 were exophytic bacteria. Forty-one isolates were categorized as both exophytes and endophytes (Fig. 2a). Three *Bacillus* species (*B. amyloliquefaciens, B. methylotrophicus* and *B. mojavensis*) were categorized as endophytic bacteria. Three bacterial species, *Ps. lurida, Ps. lutea* and *W. limnetica* were categorized as exo- and endophytic bacteria, respectively, of wild apple twigs (Fig. 2b).

**Figure 3:**
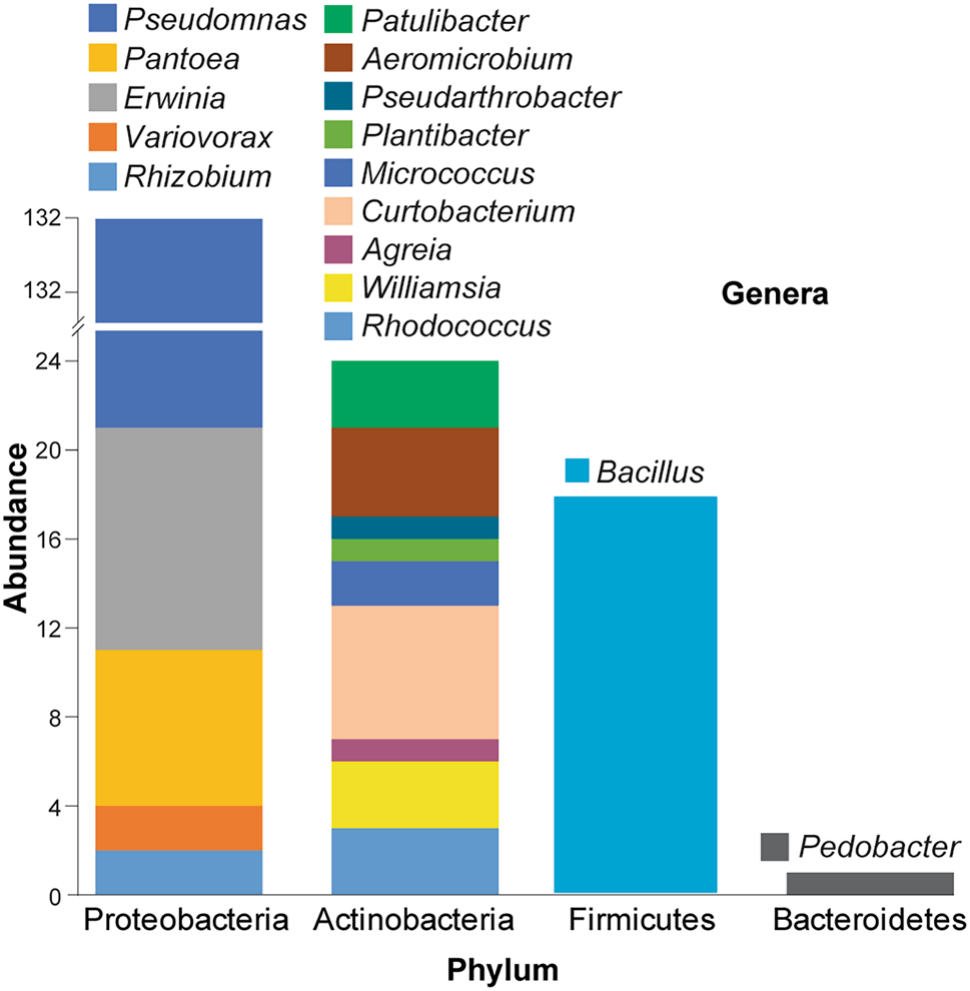
Relative OTU abundance of wild apple-associated bacteria. The abundance refers to the relative proportion of OTUs containing genera within the distribution of each parent phylum displayed on the x-axis.

BLAST analysis revealed that 37 operational taxonomic units (OTUs) (Supplementary Data S1) were distributed among four phyla, seven classes, ten orders, eleven families and thirteen genera (Table 1; Fig. 3). According to phylogenetic analysis of the sequenced bacterial species, Actinobacteria and Proteobacteria were the two largest clades, followed by Firmicutes and Bacteroidetes (Table 1; Fig. 2c). Among the Proteobacteria found in the wild apple plants, Gammaproteobacteria accounted for the largest proportion (73.1%). Within the phylum Actinobacteria, Actinomycetia was the most abundant (12.0%), and within the phylum Firmicutes, the class Bacilli accounted for 10.2% (Fig. 3).

**Table 1.** 16S-gDNA sequence analysis of bacterial isolates identified from wild apple stems and their taxonomic status.

Phylum	Class	Order	Family	Genus	Predicted species based on reference’s GenBank ID	Number of isolates
Actinobacteria	Actinomycetia	Corynebacteriales	Nocardiaceae	*Rhodococcus*	*R. cercidiphylli* NR_116275***	1
					*R yunnanensis* NR_043009***	2
				*Williamsia*	*W. limnetica* NR_117925***	2
					*W muralis* NR_037083***	1
		Micrococcales	Microbacteriaceae	*Agreia*	*A pratensis* NR_025460***	1
				*Curtobacterium*	*C. flaccumfaciens* NR_025467***	6
			Micrococcaceae	*Micrococcus*	*M. luteus* NR_075062***	1
					*M. yunnanensis* NR_116578***	1
				*Plantibacter*	*Pl. flavus* NR_025462***	1
				*Pseudarthrobacter*	*Pse. siccitolerans* NR_108849***	1
		Propionibacteriales	Nocardioidaceae	*Aeromicrobium*	*Ae. fastidiosum* NR_044983***	4
	Thermoleophilia	Solirubrobacterales	Patulibacteraceae	*Patulibacter*	*Pa. americanus* NR_042369***	3
Bacteroidetes	Sphingobacteriia	Sphingobacteriales	Sphingobacteriaceae	*Pedobacter*	*Pe. ginsenosidimutans* NR_108685**	1
Firmicutes	Bacilli	Bacillales	Bacillaceae	*Bacillus*	*B. amyloliquefaciens* NR_117946**	7
					*B. atrophaeus* NR_024689*	1
					*B. firmus* NR_112635***	1
					*B. flexus* NR_113800***	1
					*B. methylotrophicus* NR_116240***	7
					*B. mojavensis*MK764986***	1
Proteobacteria	Alphaproteobacteria	Hyphomicrobiales	Rhizobiaceae	*Rhizobium*	*Rh. smilacinae* NR_148270***	1
					*Rh. soli* NR_115996***	1
	Betaproteobacteria	Burkholderiales	Comamonadaceae	*Variovorax*	*V. boronicumulans* NR_114214***	2
	Gammaproteobacteria	Enterobacterales	Erwiniaceae	*Erwinia*	*E. billingiae* NR_104932***	10
				*Pantoea*	*P. agglomerans* NR_041978***	7
		Pseudomonadales	Pseudomonadaceae	*Pseudomonas*	*Ps. arsenicoxydans* NR_117022***	19
					*Ps. poae* MT631989***	1
					*Ps. caspiana* NR_152639***	3
					*Ps. congelans* NR_028985***	1
					*Ps. graminis* NR_026395***	16
					*Ps. helmanticensis* NR_126220***	6
					*Ps. koreensis* NR_025228***	5
					*Ps. lurida* NR_042199***	32
					*Ps. lutea* NR_029103***	7
					*Ps. orientalis* NR_024909***	5
					*Ps. silesiensis* NR_156815***	8
					*Ps. synxantha* NR_113583***	1
					*Ps. trivialis* NR_028987***	7

Asterisks (*) indicate similarities of identified bacterial isolates with references (GenBank ID of reference shown in column six). *** indicates 99–100% similarity, ** between 98 and 99%, and * between 98 and 97% similarity.

### Determination of the cellulose activities of bacteria

**Figure 4:**
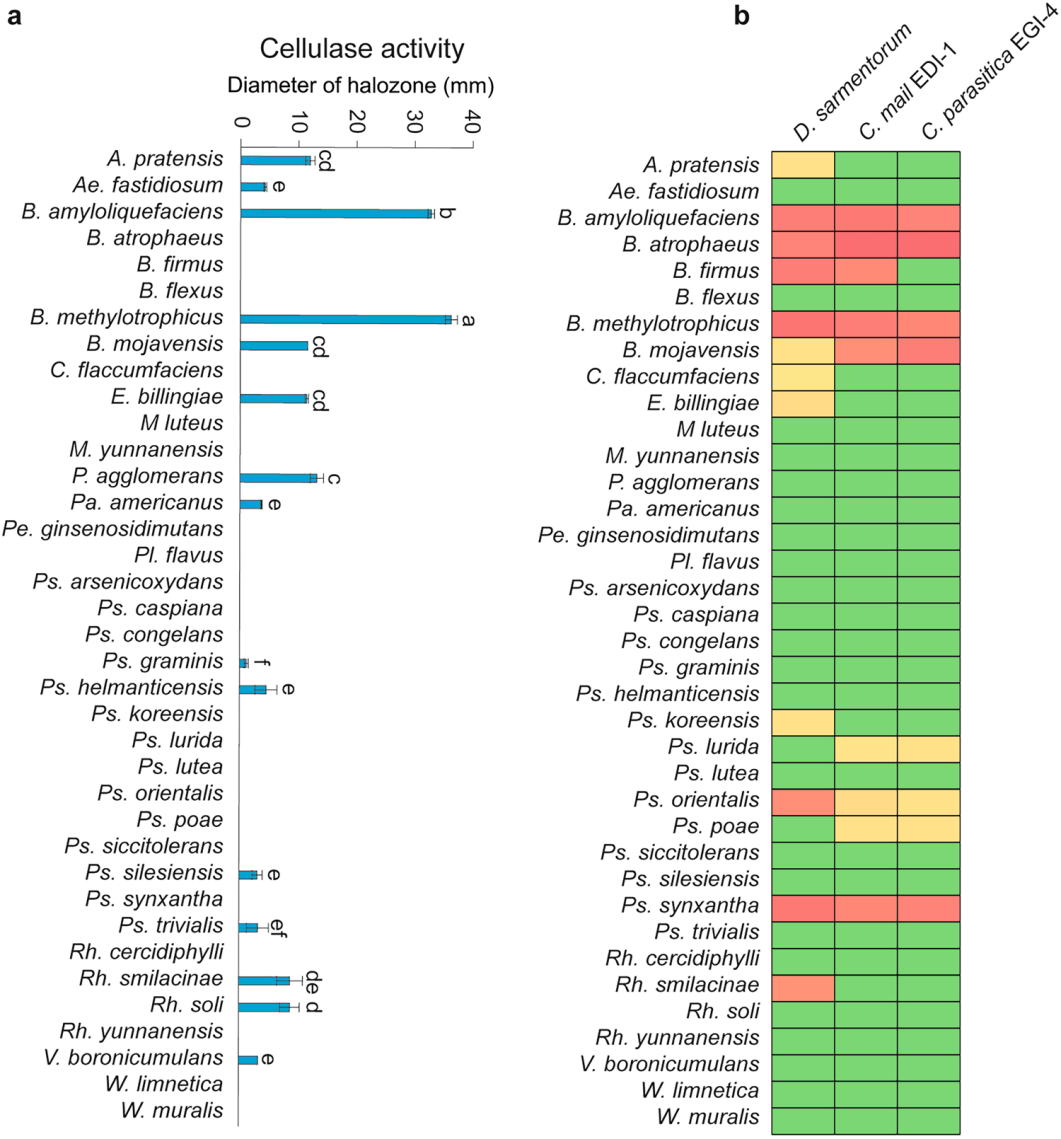
Heatmap depicting the cellulase and antifungal activities of microbial isolates obtained from wild apple twigs. Cellulase activity of isolated bacteria (**a**) and wild apple-associated bacterial species inhibited by nonpathogenic and pathogenic fungal species (**b**).

Plant cell-wall compound degradation by endophytic microorganisms occurs because it is an essential ecological process that removes dead cell wall compounds from living plants and recycles cellulose in the biosphere. We examined cultivable bacteria from a wild apple twig because of their ability to break down plant cellulosic compounds. Approximately forty percent, or 15 out of the 37 isolates, demonstrated cellulase activity to varying degrees (Fig. 4a). Cellulase activity was found to be species dependent. For example, three out of six *Bacillus* species showed the ability to break down cellulose, and among them, the strongest activity was observed in endophytic *B. amyloliquefaciens* and *B. methylotrophicus* (Fig. 4a).

### Determination of the antagonistic ability of wild apple-associated bacteria

To determine the antagonistic ability of the bacteria, the cocultivation method in dual PDA/ISP2 (50%/50%) media was used against the pathogenic fungi *C. mali*, and *C. parasitica*, and the apple-associated fungus *D. sarmentorum*. The use of dual media allows the simultaneous cultivation of fungi on PDA and bacteria on ISP2 media. The results indicated that 13 of the 37 wild apple-associated bacterial species had antagonistic effects on the pathogenic fungi *C. mali* EGI-1 and *C. cytospora* EGI-4, including the common pathogenic fungus *D. sarmentorum,* which exhibited different inhibition levels (Fig. 4b). Most of the bacterial species with the strongest antifungal activity were *Bacillus* species. Among the *Pseudomonas* species, *Ps. synxantha* exhibited the strongest antagonistic effects on all fungal species, but other *Pseudomonas* species demonstrated weak antagonistic effects. Bacterial species such as *Ps. koreensis, A. pratensis, C. flaccumfaciens, Rh. smilacinae* and *E. billingiae* also demonstrated weak antagonistic effects on *D. sarmentorum* fungi.

### A bacterial antifungal compound

Our results showed that *B. amyloliquefaciens, B. atrophaeus, B. methylotrophicus, B. mojavensis* and *Ps. synxantha* were able to strongly inhibit the growth of pathogenic fungi*.* It is known that the above-identified endophytic antagonistic *Bacillus* species produce known antifungal compounds that can inhibit the growth of different species of fungi^36–39^. Among *Pseudomonas* species, nonendophytic *Ps. synxantha* demonstrated the strongest antifungal activity against pathogenic *Cytospora* species. To better understand the antagonistic ability of *Ps. synxantha,* an analytical chemistry approach was applied to analyse antifungal compounds following our earlier work^40^. In an earlier study, we analysed antifungal compounds from the same bacterial species that were found in the guts of apple wood borer larvae. To understand whether this bacterium differed from gut bacteria, we analysed its antifungal activity.

**Figure 5:**
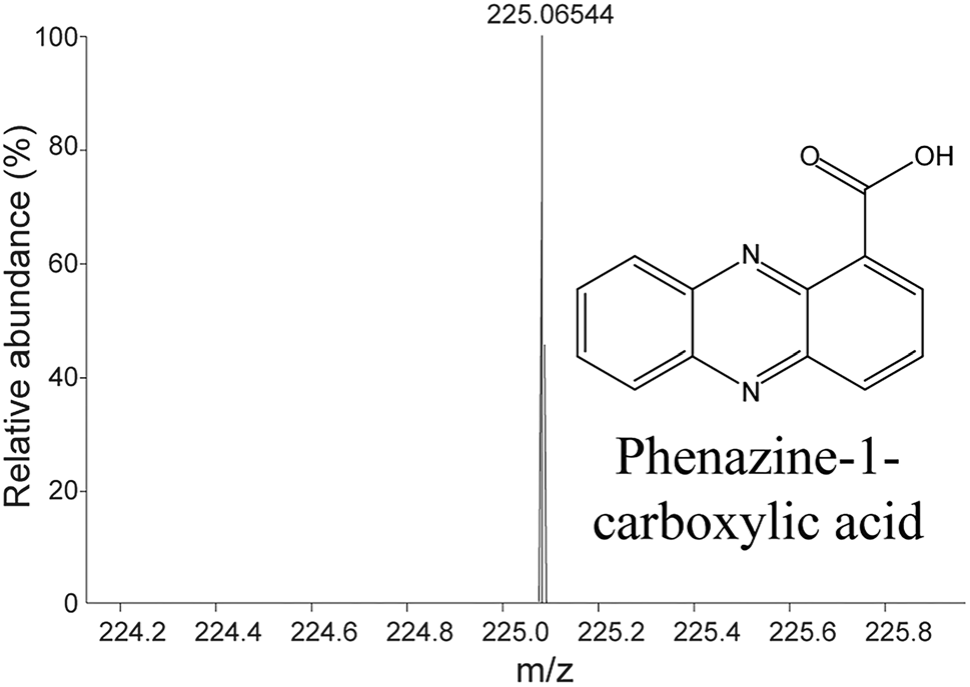
Mass spectra and chemical structure of antifungal PCA.

The crude methanolic extract from this bacterium was fractionated with silica and Sephadex gel column chromatography using optimized solvent systems. In contrast to our earlier study^40^ we excluded the petroleum:methanol mobile phase from the silica gel chromatography. The antifungal activity of each fraction was assessed via agar diffusion. The active fractions were combined and fractionated by mass via Sephadex column chromatography. Similarly, each fraction was examined again for antifungal activity. The purity of the active fraction was analysed by analytical HPLC, and the mass of each fraction was determined by mass spectrometry (Fig. 5; Supplementary Fig. S1). Next, the structure of the purified active compound was analysed by NMR (Fig. 5; Supplementary Fig. S2). Mass spectrometry and NMR analysis indicated that it was PCA from apple tree-associated bacteria, and it is not structurally different from the bacterial antifungal compound found in the larval gut^40^.

### Minimum inhibitory concentration

**Figure 6:**
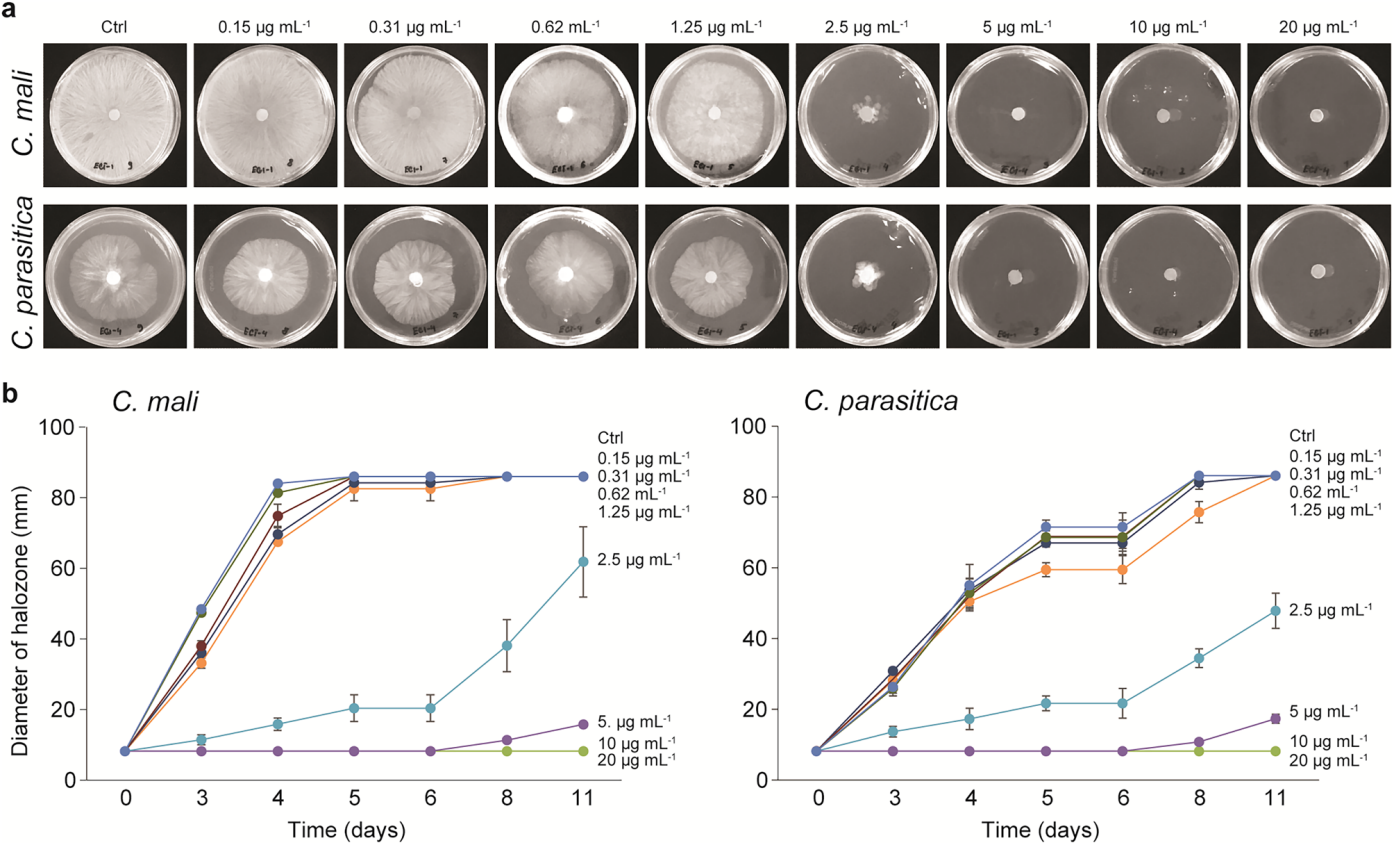
Minimum inhibitory concentration of antifungal PCA against the pathogens *C. mali* and *C. parasitica*. MICs of the antifungal compounds 6 days postcultivation on PDA plates at 28 °C (**a**). The effect of antifungal compounds at serially diluted concentrations on fungal growth at various postincubation times. The different letters indicate significant differences according to one-way ANOVA followed by a Fisher’s PLSD post hoc test (p < 0.05) (**b**).

The fractions obtained from both pathogenic fungi during purification were monitored by agar diffusion assays. To determine the MICs of the compounds for pathogenic fungi, serially diluted compounds were added to PDA agar. A purified antifungal compound at an MIC of 10 µg mL^−1^ clearly inhibited the mycelial growth of the pathogenic fungi (Fig. 6a). The effective doses of 50% and 80% of the serially diluted PCA were 2.5 µg mL^−1^ and 5 µg mL^−1^ for *C. mali* and *C. parasitica,* respectively, as estimated at 11 days postincubation (Fig. 6b). However, mycelial growth was not observed at a concentration of 5 µg mL^−1^ after 6 days of culture (Fig. 6).

## Discussion

Utilizing biological control agents against pathogenic fungi is one of the most effective strategies in agriculture^25,26,41^. Evolutionary host-associated microorganisms not only coexist mutually with each other but also develop strategies to compete with each other^42^. Microbial competition produces an extraordinary array of defensive strategies, such as antibiotics, exotoxins, volatile compounds, lytic enzymes, and other secondary metabolites, that include a wide range of defensive systems against bacteria, fungi, and higher organisms, including humans^43^. Many secondary metabolites produced by microorganisms have been reported in the literature^44^. Among the secondary metabolites produced by bacteria are antimicrobial agents^45,46^ such as peptides, terpenoids, bacteriocins, volatile compounds, lipopeptides, polyketides, and lytic enzymes. Bacterial antimicrobial metabolites are known for their ability to inhibit the growth of pathogenic fungi in plants and animals^47–49^.

Canker diseases caused by fungi are important diseases of apple trees that often result in dieback^12–17^. The primary objective of the study was to identify and screen antagonistic bacteria that could counteract the canker-causing pathogenic Cytospora species affecting wild apples. This study focused on bacteria from the same host-associated bacteria found in the Tianshan wild apple forest in Western China. More than 175 bacterial isolates were obtained from wild apple twigs and screened for their antifungal ability against pathogenic *Cytospora* fungal species using in vitro dual coculture. Eight bacterial isolates belonging to the genera *Bacillus* and *Pseudomonas* exhibited antifungal activity against the canker-causing pathogens *C. mali*, *C. parasitica*, and *D. sarmentorum*. Among them, four *Bacillus* isolates and one *Ps. synxantha* isolate exhibited stronger antifungal effects on pathogenic fungi than did other bacteria. The results suggest that these antagonistic bacteria should be further studied for their biological control ability in vivo against pathogenic fungi.

A number of studies have reported that the abovementioned *Bacillus* species produce antifungal metabolites against fungi^50–52^. It has been shown that *B. amyloliquefaciens* inhibits the growth of the pathogenic fungus *Cytospora pistaciae*^51^ by producing volatile organic compounds (VOCs)^53–55^. Similarly, *B. atrophaeus* and *B. methylotrophicus* have also been shown to inhibit a wide range of pathogenic fungi, including *Cytospora* species^56,57^ by producing VOCs and lipopeptides^58,59^. Commercially available antagonistic *B. mojavensis* is known for its antifungal ability against the pathogen *Diplodia corticola,* which causes canker disease in oak trees^60^, by producing the abovementioned active compounds^61,62^. Our results also consistently demonstrated that apple-associated *Bacillus* species inhibited the canker-causing *Cytospora* species. Most likely, the *Bacillus* species examined in this study could produce known antifungal VOCs since the molecular identification of bacterial isolates revealed that they belonged to *B. amyloliquefaciens*, *B. atrophaeus, B. methylotrophicus,* and *B. mojavensis*. In addition, these *Bacillus* species are known to be endophytes^61,63,64^ which is consistent with our finding that they colonize the inside of the tree. These bacterial isolates show promise for being utilized as biological control agents against canker-causing fungal pathogens because of their ability to colonize plant organs.

*Pseudomonas* species are known for being beneficial plant growth-promoting bacteria, and they are used as biocontrol agents^65,66^ due to the production of various types of antifungal compounds^67,68^. This genus showed varying levels of antifungal activity against pathogenic *Cytospora* fungi. Among the fourteen identified *Pseudomonas* species, only *Ps. synxantha* demonstrated the strongest antifungal activity, whereas *Ps. orientalis*, *Ps. poae*, and *Ps. lurida* had weak antagonistic effects. Many studies have reported that *Pseudomonas* species have antifungal effects on pathogenic fungi, and several members have been used for commercial purposes as biofungicides, which synthesize antifungals^69^. Species of the genus *Pseudomonas* can produce various types of antifungal metabolites such as 2,4-diacetylphloroglucinol, phenazines, pyrrolnitrin, pyoluteorin, oomycin A, agrocin 84, hydrogen cyanide, and pseudobactin B10, as well as the complex macrocycliclactone 2,3-de-epoxy-2,3-didehydro-rhizoxin^70–73^. A chapter in a review book by Höfte listed ten *Pseudomonas* species that are used as bacterial biocontrol agents to control plant disease^74^. In an earlier study^40^ and in this study, we found another *Pseudomonas* sp. that had 99.51% similarity with *Ps. synxantha*. Active PCA from *Ps. synxantha* extracted using an optimized protocol at a concentration of 2.5 µg mL^−1^ inhibited the in vitro mycelial growth of *C. mali* and *C. parasticia* by 55% and 60%, respectively; therefore, this concentration was more effective than that used against pathogenic *Botrytis cinerea*^75^. This indicated that the two *Cytospora* species were more sensitive to PCA and could be used for the control of canker disease. To our knowledge, this is the first report in which *Pseudomonas* species or their active metabolites were tested against canker-causing pathogenic *Cytospora* fungi.

Furthermore, in our previous study, we examined the antifungal ability of the larval gut bacteria of the invasive apple borer *Agrilus mali,* which prevents fungal colonization of the gut in an invaded region of the Tianshan Forest^40,76^. The gut of this larva is reportedly normally colonized by fungi^77^. The gut bacteria *Ps. synxantha* prevented fungal colonization of the gut by producing the antifungal compound PCA^40^. This finding also validated that *Ps. synxantha,* with its similar genetics and antifungal compounds found in larval guts, originated from apple microflora.

Overall, we identified and examined cultivable apple-associated bacteria for their antagonistic ability against the canker-causing pathogens *Cytospora* species, *C. mali,* and *C. parasitica*. Among the isolated bacteria, four *Bacillus* and *Pseudomonas* species were revealed to be strong antagonists of canker-causing fungi. Since the examined *Bacillus* species produce known antimicrobial metabolites, *Ps. synxantha* was used for the determination of antifungal compounds. The antifungal compound PCA, extracted from *Ps. synxantha* demonstrated high efficiency at low concentrations in vitro against pathogenic fungi and may have potential for use in controlling apple canker disease. However, to validate the inhibition efficiency, either antagonistic bacteria or their antifungal effects on plants inoculated with pathogenic fungi will be studied in future experiments.

## Materials and methods

### Plant, fungi, and bacterial species

Three wild apple twigs were randomly collected from a wild apple nursery in Mohe Village (43° 51 N, 82°15 E), located in the Ili Valley of the Tianshan Mountains, Xinjiang-Uyghur Autonomous Province, China, for bacterial isolation. The two pathogenic fungal species, *Cytospora mali* EGI-1 and *C. parasitica* EGI-4, used in this study were obtained from our previous work^18^ and another canker-causing pathogenic fungus *Dothiorella sarmentorum*^78^, which is a common dominant species in wild apple mycoflora from our earlier study^40^, was used as a control.

The use of plant material compiled according to the relevant guidelines and legislation. We received permission from the Yili Forest and Grass Bureau of Xinjiang-Uyghur Autonomous Province, China, before the collection of wild apple twigs.

### Bacterial isolation and identification

Wild apple twig-associated bacteria (exo- and endophytes) were isolated following the methods described in our previous works^40,76^. The apple twig surface was sterilized with 95% ethyl alcohol for 3 min and washed three times with sterile water. Furthermore, sterilized twigs were homogenized with a sterilized homogenizer under aseptic conditions for endophytic bacterial isolation. Nonsterilized twigs were also homogenized to isolate both exo- and endophytic bacteria. The homogenates from sterilized and nonsterilized twigs were incubated in PBS buffer (NaCl—137 mM, KCl—2.7 mM, Na_2_HPO_4_—1 mM, KH_2_PO_4_—1.8 mM; pH 7.4) and serially diluted to 10^−6^ by the addition of sterilized buffer. Each diluted sample was spread on nutrient agar (NA) (0.5% peptone, 0.3% beef extract, 1.5% agar, pH 6.8) (Difco, France) in a laminar flow cabinet. The plates were kept at 28 °C for 48–96 h in a thermostatic incubator upon the appearance of bacterial colonies. Furthermore, single colonies were randomly selected based on colony features (colony size, shape, colour, elevation, margin, opacity, and consistency).

### Isolation of antagonistic bacteria

To select antagonistic bacteria associated with apple twigs against pathogenic *C. mali* or *C. parasitica* fungi, bacteria and fungi were cocultivated in mixed agar media that contained half potato dextrose agar (PDA) (potato starch 4 g L^−1^, dextrose 20 g L^−1^, and agar 15 g L^−1^, pH 7.2) (Beijing, Solarbio) and half ISP2 agar (yeast extract 4 g L^−1^, malt extract 10 g L^−1^, dextrose 4 g L^−1^, agar 20 g L^−1^, pH 7.2). Punched-out 0.7 mm-diameter PDA gel pieces from a fungus-cultured agar plate were transferred onto the middle of a mixed agar medium plate, and mycelial growth was evaluated 3, 4, 5, 6, 8, and 11 days postincubation at 25 °C. The distance between the bacterial growth edge and the fungal growth edge was calculated to determine the extent of mycelial inhibition by antagonistic bacteria. Fungal mycelial growth inhibition was estimated using the following equation, as described by Alenezi et al.^79^:

$$ {\text{I }}\left( \% \right) \, = \, \left( {{1} - {\text{a}}/{\text{b}}} \right)*{1}00 $$

where *a* is the distance from the centre of the fungal colony to the fungal growth edge on the bacterial side, and *b* is the radius of control of the fungal colony.

### Bacterial DNA extraction and PCR amplification

DNA extraction was carried out following the method described in our earlier work^76^. Briefly, bacterial colonies were cultured in 2 mL of LB broth (10 g/L tryptone, 5 g/L yeast extract, and 5 g/L NaCl, pH 7.0) on a shaker incubator at 250 rpm at 28 °C overnight. Bacterial DNA was extracted using a TIANamp Bacteria DNA Kit (Tiangen, China) following the manufacturer's protocol. The primer pairs 27F (5′-AGAGTTTGATCATGGCTCAG-3′) and 1492R (5′-TACGGCTACCTTGTTACGACTT-3′) were used for PCR amplification on a Veriti thermocycler (Applied Biosystems, United States). Amplifications were performed in 50 μL containing 10 μL of ready-to-use PrimeSTAR HS (Premix) (Takara, Japan) master mix, 1 μL (0.2 μM) of each primer, and 1 μL of DNA. The PCR conditions were as follows: 5 min at 95 °C for the initial denaturation step; 35 cycles of denaturation at 94 °C for 15 s, annealing at 55 °C for 30 s, and elongation at 72 °C for 2 min; and a final extension at 72 °C for 10 min. PCR products were visualized on a 1.5% agarose gel. The PCR products were purified and sequenced bidirectionally using the Sanger method at QuintaraBio (China).

### Sequence analysis

SeqMan (DNASTAR Lasergene 7) was used for sequence assembly. Bacterial 16S-rDNA sequences were compared with those of respective bacterial species deposited in GenBank using the BLASTN algorithm. Sequences with high identity were scored. Furthermore, these sequences were aligned with CLUSTALW (MEGA7). A maximum likelihood (ML) phylogenetic tree was built using the neighbour-joining algorithm following the Tajima–Nei model with 1000 bootstrap replicates in MEGA7.

### Cellulase activity

Cellulase activity was assessed using carboxymethylcellulose-containing medium supplemented with 7.5 g/L carboxymethylcellulose, 1.6 g/L KCl, 1.43 g/L NaCl, 0.15 g/L NH_4_Cl, 0.037 g/L MgSO_4_·7H_2_O, 0.94 g/L KH_2_PO_4_, 1.9 g/L K_2_HPO_4_, 0.017 g/L CaCl_2_, 0.1 g/L yeast extract, and 15 g/L agar, pH 7.0 as described by Vasanthakumar et al.^80^. Bacterial isolates were cultured on media for 96 h, where carboxymethylcellulose served as the sole carbon source. After incubation, the colonies were rinsed with water. Congo red solution (0.5%) was used for agar plate staining for 30 min until the carboxymethylcellulose became dye-bound. Next, to fix the colouration, the plates were rinsed with 1 M NaCl for 5 min and then washed with water. Halos were measured with a calliper.

### Extraction and purification of antifungal compounds

To isolate an antifungal compound from bacteria, we followed the optimized protocol from previous work with some modifications^40^. Bacteria were cultured in 2 L of LB liquid medium, and after 5 days, the culture was centrifuged at 8000 rpm for 10 min to obtain cell-free supernatants. The supernatants were dehydrated under a fume hood and extracted with methanol. Next, these extracts were vortexed and centrifuged at 10,000 rpm for 5 min. The supernatants were concentrated in a rotary evaporator (IKA RV8V, Germany), and the contents were dissolved in 1 mL of methanol.

Silica gel column chromatography was used to fractionate the crude extract (14 g). Equal amounts of silica gel (200–300 mesh) (Qingdao Marine Chemical Company, China) were mixed with the crude extract and loaded at the top of a chromatography column (80 cm in length and 5 cm in diameter) containing 140 g of silica gel. Silica gel column fractionation was performed with different solvent combinations of hexane:ethyl acetate (9:1, 4:1, 2:1, 1:1, and 0:1 v/v) and methanol: dichloromethane (100:0, 36:1, 18:1, 9:1, 4:1, 2:1, 1:1, and 0:100 v/v). The antifungal activity of each fraction was examined via agar diffusion. Furthermore, active fractions were merged, and based on thin-layer chromatography (TLC) of compound separation, Sephadex column-based fractionation (50 cm length and 1.5 cm diameter) (Sephadex LH-20, Amersham Pharmacia Biotech, Sweden) was performed with a mobile phase of combinations of dichloromethane and methanol (100:0, 70:1, 0:100). The antifungal activity of each fraction was analysed by agar diffusion and TLC. The active fractions were analysed by HPLC using a Hitachi Chromaster HPLC system consisting of a 1,110 pump, a DT-230 column oven, a 1,430 diode array detector, and a YMC C18 column (250 × 4.6 mm, 5 mm). Nuclear magnetic resonance (NMR) analysis was performed following the method described in our earlier study^40^.

To determine the antifungal activity of the fractions from the silica and Sephadex columns by chromatography, holes 3 mm in diameter were made in PDA plates with a hole puncher. Each fraction was loaded into the well under sterile conditions. Next, a 5 mm diameter fungal mycelial disc grown on a PDA plate was punched out and placed in the middle of the PDA plate, where it was cultured for 7 days at 25 °C. The antifungal activity of the fractions was recorded based on fungal growth performance.

### Thin-layer chromatography, HPLC, mass spectrometry, and NMR analyses

Qualitative and quantitative analyses of antifungal compounds were performed using TLC, HPLC, mass spectrometry, and nuclear magnetic resonance methods, as described in our earlier work^40^.

### Minimum inhibitory concentration (MIC)

To determine the MIC for the fungi *C. mali* and *C. parasitica*, a purified 20 µg/mL antifungal compound was dissolved in 5% DMSO. Next, the solution was diluted by the addition of an equal amount of 5% DMSO to achieve concentrations of 20, 10, 5, 2.5, 1.25, 0.63, 0.31, and 0.16 mg mL^−1^ concentrations. PDA medium was prepared and cooled to 55 °C, and the diluted compounds at the indicated concentrations were mixed with the medium to prepare PDA plates. The punched-out gel disc from the pathogenic fungal culture with a 5-mm diameter was transferred onto the middle of the PDA plate. Mycelial growth was evaluated after 3, 4, 5, 6, 8, and 11 days of incubation at 25 °C. DMSO (5%) without compound was used as a control. All treatments were performed in triplicate. Mycelial growth inhibition (MGI) was determined with callipers, and MIG was calculated using the following equation:

$${\text{MGI}}=\left[\frac{{\text{Dc}}-{\text{Dt}}}{{\text{Dc}}}\right]\mathrm{x }100$$

where Dc (mm) is the mean colony diameter in the control, and Dt (mm) is the mean colony diameter in each treatment.

### Statistical analysis

All the experimental data are presented as the means of at least three independent replicates, and comparisons of the data were performed using one-way ANOVA with Fisher’s PLSD post hoc test. A p-value of < 0.05 was considered to indicate statistical significance. All the statistical analyses were performed using StatView software packages (Version 5.0.1, SAS Institute Inc., Cary, NC, United States, http://www.statview.com). All figures were generated in Adobe Illustrator CS3, version 13.0.0.

### Supplementary Information

Supplementary Information.

Supplementary Figure S1.

Supplementary Figure S2.

Supplementary Legends.

## Data Availability

The bacterial 16S gDNA generated during the current study are available in GenBank (http://www.ncbi.nlm.nih.gov) under the accession numbers PP237222, PP237223, PP237224, PP237225, PP237226, P237227, PP237228, PP237229, PP237230, PP237231, PP237232, PP237233, PP237234, PP237235, PP237236, PP237237, PP237238, PP237239, PP237240, PP237241, PP237242, PP237243, PP237244, PP237245, PP237246, PP237247, PP237248, PP237249, PP237250, PP237251, PP237252, PP237253, PP237254, PP237255, PP237256, PP237257 and PP237258.

## References

[1] Cornille A, Giraud T, Smulders MJ, Roldan-Ruiz I, Gladieux P (2014). The domestication and evolutionary ecology of apples. Trends Genet..

[2] Duan N (2017). Genome re-sequencing reveals the history of apple and supports a two-stage model for fruit enlargement. Nat. Commun..

[3] Lin, P. & Lin, Y. In *Wild Fruit Forests Resources in Tianshan Mountains-Comprehensive Research on Wild Fruit Forests in Ili, Xinjiang, China* (eds P. Lin & N. Cui) 46–62 (China Forestry Publishing House, 2000).

[4] Richards CM (2009). Genetic diversity and population structure in *Malus **sieversii*, a wild progenitor species of domesticated apple. Tree Genet. Genomes.

[5] Dean R (2012). The Top 10 fungal pathogens in molecular plant pathology. Mol. Plant Pathol..

[6] O’Brien PA (2017). Biological control of plant diseases. Australas. Plant Pathol..

[7] Fisher MC (2012). Emerging fungal threats to animal, plant and ecosystem health. Nature.

[8] Liu AH (2018). Canker and fine-root loss of *Malus **sieversii* (Ldb.) Roem. caused by *Phytophthora **plurivora* in Xinjiang Province in China. Forest Pathol..

[9] Ji Y, Ji R, Huang RX (2004). Invasive species—*Agrilus mali* Matsumura and damage in Xinjiang. Xinjiang Agric. Sci..

[10] Bozorov TA, Luo Z, Li X, Zhang D (2019). *Agrilus mali* Matsumara (Coleoptera: Buprestidae), a new invasive pest of wild apple in western China: DNA barcoding and life cycle. Ecol. Evol..

[11] Wang, Z. Y. *Research on biological control of Agrilus mali Matsumura (Coleoptera: Buprestidae) in stands of Malus sieversii in Xinjiang.* Doctor thesis, Chinese Academy of Forestry (2013).

[12] Brown-Rytlewski DE, McManus PS (2000). Virulence of *Botryosphaeria **dothidea* and *Botryosphaeria **obtusa* on apple and management of stem cankers with fungicides. Plant Dis..

[13] Zhang Q (2014). Induction of resistance mediated by an attenuated strain of *Valsa mali* var. mali using pathogen-apple callus interaction system. ScientificWorldJournal.

[14] Abe K, Kotoda N, Kato H, Soejima J (2007). Resistance sources to Valsa canker (*Valsa **ceratosperma*) in a germplasm collection of diverse *Malus* species. Plant Breed..

[15] Wang X, Wei J, Huang L, Kang Z (2011). Re-evaluation of pathogens causing Valsa canker on apple in China. Mycologia.

[16] Wang X, Zang R, Yin Z, Kang Z, Huang L (2014). Delimiting cryptic pathogen species causing apple Valsa canker with multilocus data. Ecol. Evol..

[17] Nourian A, Salehi M, Safaie N, Khelghatibana F, Abdollahzadeh J (2021). Fungal canker agents in apple production hubs of Iran. Sci. Rep..

[18] Liu X (2020). Characterization and pathogenicity of six Cytospora strains causing stem canker of wild apple in the Tianshan Forest, China. For. Pathol..

[19] Slippers B (2004). Multiple gene sequences delimit *Botryosphaeria australis* sp. nov. from *B. lutea*. Mycologia.

[20] Phillips AJ, Lopes J, Abdollahzadeh J, Bobev S, Alves A (2012). Resolving the Diplodia complex on apple and other Rosaceae hosts. Persoonia.

[21] Cloete M, Fourie PH, Damm U, Crous PW, Mostert L (2011). Fungi associated with die-back symptoms of apple and pear trees, a possible inoculum source of grapevine trunk disease pathogens. Phytopathol. Mediterr..

[22] Wang C (2014). Toxins produced by *Valsa mali* var. mali and their relationship with pathogenicity. Toxins.

[23] Biggs AR, El-Kholi MM, ElNeshawy SM (1994). Effect of calcium salts on growth, pectic enzyme activity, and colonization of peach twigs by *Leucostoma** persoonia*. Plant Dis..

[24] Biggs AR, Grove GG (2005). Leucostoma canker of stone fruits. Plant Health Instruct..

[25] Thambugala KM, Daranagama DA, Phillips AJL, Kannangara SD, Promputtha I (2020). Fungi vs. fungi in biocontrol: An overview of fungal antagonists applied against fungal plant pathogens. Front. Cell Infect. Microbiol..

[26] Hostettmann K, Wolfender JL (1997). The search for biologically active secondary metabolites. Pest Manag. Sci..

[27] Wilson CL (1991). Biological control of post-harvest diseases of fruits and vegetables: Alternatives to synthetic fungicides. Crop Protect..

[28] Wisniewski M, Droby S, Norelli J, Liu J, Schena L (2016). Alternative management technologies for postharvest disease control: The journey from simplicity to complexity. Postharvest Biol. Technol..

[29] Droby S (2006). Biological control of postharvest diseases of fruits and vegetables: Difficulties and challenges. Phytopathol. Pol..

[30] Hoyos-Villegas V, Chen J, Mastrangelo AM, Raman H (2022). Editorial: Advances in breeding for quantitative disease resistance. Front. Plant Sci..

[31] Alabouvette C, Olivain C, Migheli Q, Steinberg C (2009). Microbiological control of soil-borne phytopathogenic fungi with special emphasis on wilt-inducing *Fusarium **oxysporum*. New Phytol..

[32] Debbi A, Boureghda H, Monte E, Hermosa R (2018). Distribution and genetic variability of *Fusarium **oxysporum* associated with tomato diseases in Algeria and a biocontrol strategy with indigenous *Trichoderma* spp. Front. Microbiol..

[33] Gomez-Lama Cabanas C (2018). Indigenous *Pseudomonas* spp. strains from the olive (*Olea europaea* L.) Rhizosphere as effective biocontrol agents against *Verticillium **dahliae*: From the host roots to the bacterial genomes. Front. Microbiol..

[34] Guardado-Valdivia L (2018). Identification and characterization of a new Bacillus atrophaeus strain B5 as biocontrol agent of postharvest anthracnose disease in soursop (*Annona muricata*) and avocado (*Persea** americana*). Microbiol. Res..

[35] Mefteh FB (2017). Fungal root microbiome from healthy and brittle leaf diseased date palm trees (*Phoenix dactylifera* L.) reveals a hidden untapped arsenal of antibacterial and broad spectrum antifungal secondary metabolites. Front. Microbiol..

[36] Lee HA, Kim JH (2012). Isolation of *Bacillus **amyloliquefaciens* strains with antifungal activities from Meju. Prev. Nutr. Food Sci..

[37] Rajaofera MJN (2019). Volatile organic compounds of *Bacillus **atrophaeus* HAB-5 inhibit the growth of *Colletotrichum **gloeosporioides*. Pestic. Biochem. Physiol..

[38] Cheng X (2019). Characterization of antagonistic *Bacillus **methylotrophicus* isolated from rhizosphere and its biocontrol effects on maize stalk rot. Phytopathology.

[39] Ma Z, Hu J (2014). Production and characterization of Iturinic lipopeptides as antifungal agents and biosurfactants produced by a marine *Pinctada **martensii*-derived *Bacillus **mojavensis* B0621A. Appl. Biochem. Biotechnol..

[40] Bozorov TA (2021). Wild apple-associated fungi and bacteria compete to colonize the larval gut of an invasive wood-borer *Agrilus mali* in Tianshan forests. Front. Microbiol..

[41] Pandit MA (2022). Major biological control strategies for plant pathogens. Pathogens.

[42] Prosser JI (2007). The role of ecological theory in microbial ecology. Nat. Rev. Microbiol..

[43] Riley MA, Wertz JE, Goldstone C (2004). The ecology and evolution of microbial defense systems in *Escherichia coli*. EcoSal Plus.

[44] Mousa WK, Raizada MN (2013). The diversity of anti-microbial secondary metabolites produced by fungal endophytes: An interdisciplinary perspective. Front. Microbiol..

[45] Berdy J (2005). Bioactive microbial metabolites. J. Antibiot..

[46] Coleman JJ, Ghosh S, Okoli I, Mylonakis E (2011). Antifungal activity of microbial secondary metabolites. PLoS ONE.

[47] Devi S (2019). Depiction of secondary metabolites and antifungal activity of *Bacillus **velezensis* DTU001. Synth. Syst. Biotechnol..

[48] Mishra B (2021). Antifungal metabolites as food bio-preservative: Innovation, outlook, and challenges. Metabolites.

[49] Haas D, Defago G (2005). Biological control of soil-borne pathogens by fluorescent pseudomonads. Nat. Rev. Microbiol..

[50] Zubir I, Ross EER, Hamzah A, Aqma WS (2019). Endophytic bacteria from *Theobroma cacao* L. with antifungal activities against *Phytophthora **palmivora*. Asian J. Agric. Biol..

[51] Gusella G, Vitale A, Polizzi G (2022). Potential role of biocontrol agents for Sustainable management of fungal pathogens causing canker and fruit rot of pistachio in Italy. Pathogens.

[52] Lim KB (2016). Isolation and characterization of a broad spectrum bacteriocin from *Bacillus **amyloliquefaciens* RX7. Biomed. Res. Int..

[53] Yuan J, Raza W, Shen Q, Huang Q (2012). Antifungal activity of *Bacillus **amyloliquefaciens* NJN-6 volatile compounds against *Fusarium **oxysporum* f. sp. *cubense*. Appl. Environ. Microbiol..

[54] Zhang J (2015). *Bacillus **amyloliquefaciens* GB1 can effectively control apple valsa canker. Biol. Control.

[55] Qian J (2021). Biocontrol of citrus canker with endophyte *Bacillus **amyloliquefaciens* QC-Y. Plant Prot. Sci..

[56] Huang H (2015). Identification and characterization of the endophytic bacterium *Bacillus **atrophaeus* XW2, antagonistic towards *Colletotrichum **gloeosporioides*. Ann. Microbiol..

[57] Shan H (2013). Biocontrol of rice blast by the phenaminomethylacetic acid producer of *Bacillus **methylotrophicus* strain BC79. Crop Prot..

[58] Zhang X (2013). Lipopeptides, a novel protein, and volatile compounds contribute to the antifungal activity of the biocontrol agent *Bacillus **atrophaeus* CAB-1. Appl. Microbiol. Biotechnol..

[59] He CN, Ye WQ, Zhu YY, Zhou WW (2020). Antifungal activity of volatile organic compounds produced by *Bacillus **methylotrophicus* and *Bacillus thuringiensis* against five common spoilage fungi on loquats. Molecules.

[60] Muñoz-Adalia EJ, Meijer A, Campillo-Brocal JC, Colinas C (2021). Antagonistic effect in vitro of three commercial strains of *Bacillus* sp. against the forest pathogen *Diplodia*
*corticola*. For. Pathol..

[61] Jasim B, Sreelakshmi S, Mathew J, Radhakrishnan EK (2016). Identification of endophytic *Bacillus **mojavensis* with highly specialized broad spectrum antibacterial activity. 3 Biotech..

[62] Ghazala I, Chiab N, Saidi MN, Gargouri-Bouzid R (2022). Volatile organic compounds from *Bacillus **mojavensis* I4 promote plant growth and inhibit phytopathogens. Physiol. Mol. Plant Pathol..

[63] Uwaremwe C (2021). An endophytic strain of *Bacillus **amyloliquefaciens* suppresses *Fusarium **oxysporum* infection of chinese wolfberry by altering its rhizosphere bacterial community. Front. Microbiol..

[64] Jiang Y (2019). Purification and characterization of a novel antifungal flagellin protein from endophyte *Bacillus **methylotrophicus* NJ13 against *Ilyonectria** robusta*. Microorganisms.

[65] Qessaoui R (2019). Applications of new *Rhizobacteria pseudomonas* isolates in agroecology via fundamental processes complementing plant growth. Sci. Rep..

[66] Castro Tapia MP (2020). Antagonistic activity of Chilean strains of *Pseudomonas **protegens* against fungi causing crown and root rot of wheat (*Triticum aestivum* L.). Front. Plant Sci..

[67] Jayaswal RK (1993). Antagonism of *Pseudomonas **cepacia* against phytopathogenic fungi. Curr. Microbiol..

[68] Kron AS (2020). *Pseudomonas **orientalis* F9 pyoverdine, safracin, and phenazine mutants remain effective antagonists against *Erwinia **amylovora* in apple flowers. Appl. Environ. Microbiol..

[69] Weller DM (2007). *Pseudomonas* biocontrol agents of soilborne pathogens: Looking back over 30 years. Phytopathology.

[70] Ligon JM (2000). Natural products with antifungal activity from *Pseudomonas* biocontrol bacteria. Pest Manag. Sci..

[71] Zhang Y (2019). Volatile organic compounds produced by *Pseudomonas **chlororaphis* subsp. *aureofaciens* SPS-41 as biological fumigants to control *Ceratocystis fimbriata* in postharvest sweet potatoes. J. Agric. Food Chem..

[72] Russo A (1996). Improved delivery of biocontrol *Pseudomonas* and their antifungal metabolites using alginate polymers. Appl. Microbiol. Biotechnol..

[73] Wasi S, Tabrez S, Ahmad M (2013). Use of *Pseudomonas* spp. for the bioremediation of environmental pollutants: A review. Environ. Monit. Assess..

[74] Höfte, M. In *Microbial Bioprotectants for Plant Disease Management* (eds Köhl, J. & Ravensberg, W. J.) (Burleigh Dodds, 2021).

[75] Simionato AS (2017). The effect of phenazine-1-carboxylic acid on mycelial growth of *Botrytis cinerea* produced by *Pseudomonas aeruginosa* LV strain. Front. Microbiol..

[76] Bozorov TA, Rasulov BA, Zhang D (2019). Characterization of the gut microbiota of invasive *Agrilus mali* Matsumara (Coleoptera: Buprestidae) using high-throughput sequencing: Uncovering plant cell-wall degrading bacteria. Sci. Rep..

[77] Zhang ZQ, Jiao S, Li XH, Li ML (2018). Bacterial and fungal gut communities of *Agrilus mali* at different developmental stages and fed different diets. Sci. Rep..

[78] Iqbal S (2026). *Dothiorella*
*sarmentorum* causing canker and branch dieback of English walnut in Maule region, Chile. Plant Dis..

[79] Alenezi FN (2016). Strain-level diversity of secondary metabolism in the biocontrol species *Aneurinibacillus*
*migulanus*. Microbiol. Res..

[80] Vasanthakumar A, Handelsman JO, Schloss PD, Bauer LS, Raffa KF (2008). Gut microbiota of an invasive subcortical beetle, *Agrilus **planipennis* Fairmaire, across various life stages. Environ. Entomol..

