# Supplementary material for: Uncovering the antifungal activities of wild apple-associated bacteria against two canker-causing fungi, Cytospora mali and C. parasitica
Source: Sci Rep. 2024 Mar 15;14:6307. doi: 10.1038/s41598-024-56969-4 (PMC10943224; doi:10.1038/s41598-024-56969-4)
Supplement: Supplementary file 2 — Supplementary Figure S1. [file 41598_2024_56969_MOESM2_ESM.pdf]

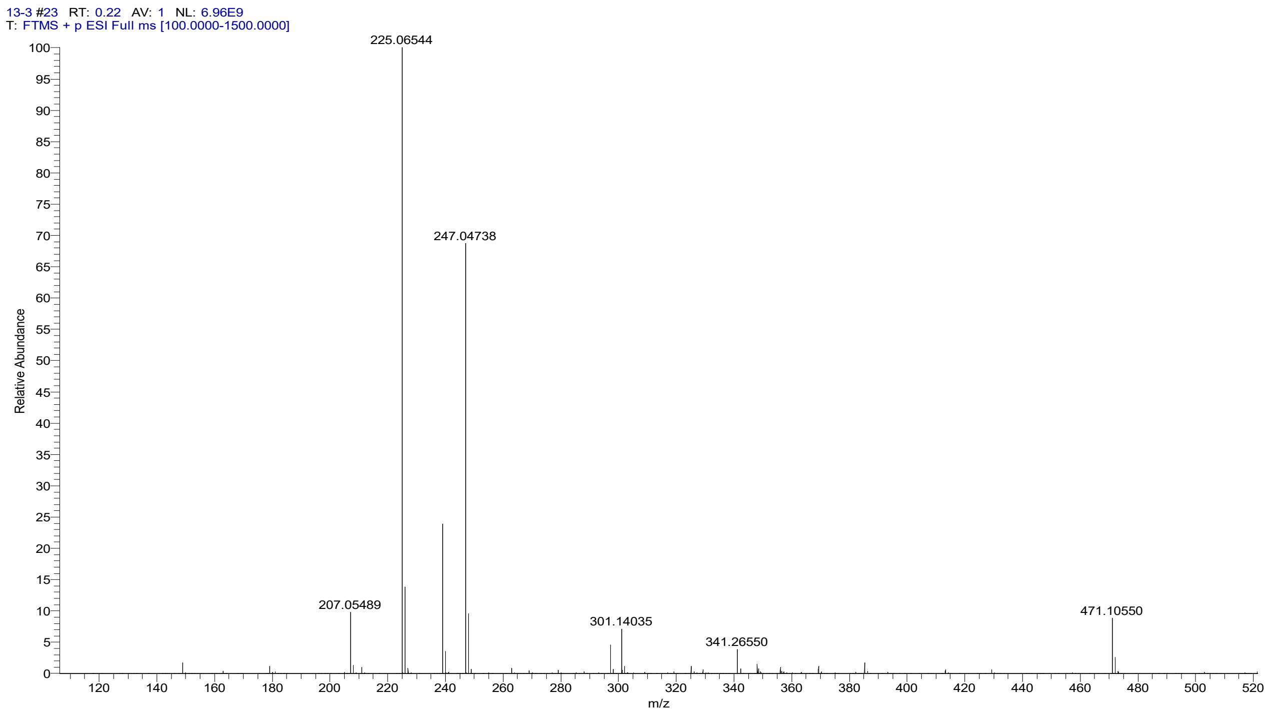

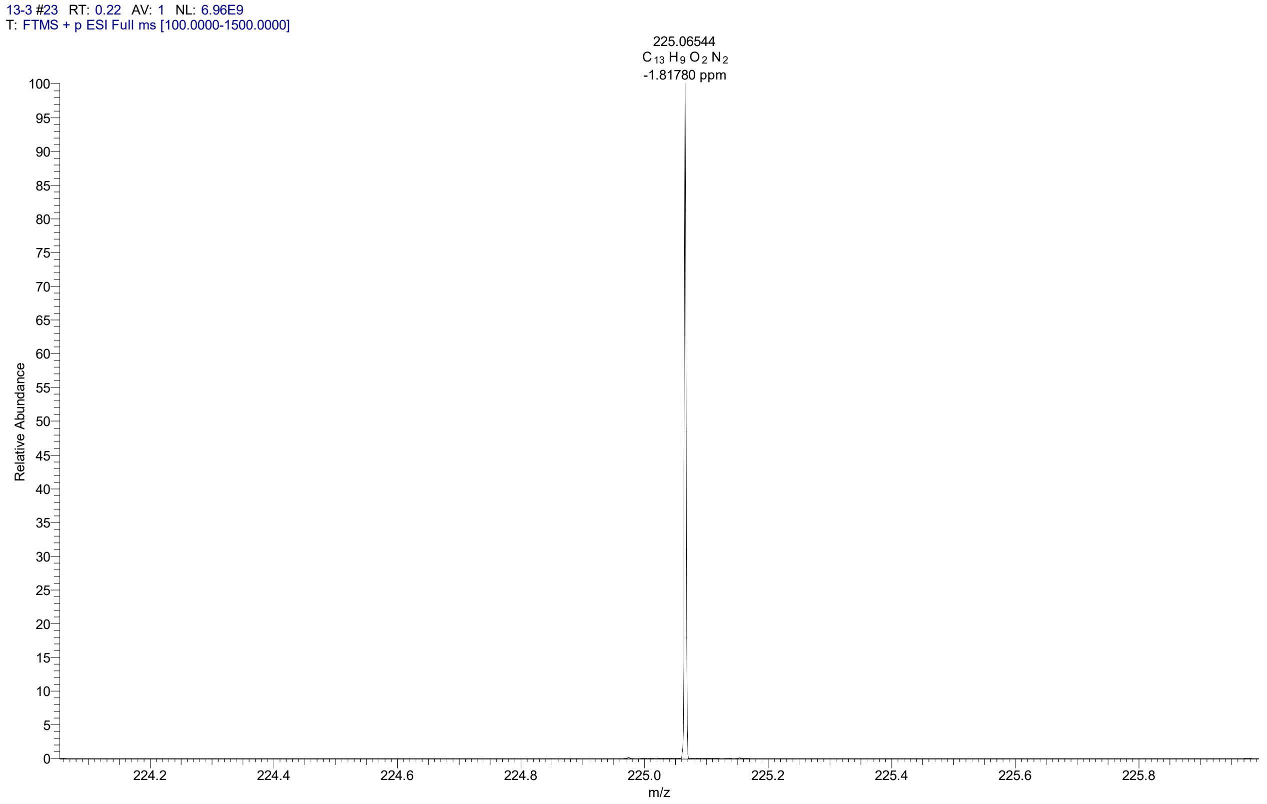

Supplementary figure S1. Mass spectrometry mass
