# Supplementary material for: Uncovering the antifungal activities of wild apple-associated bacteria against two canker-causing fungi, Cytospora mali and C. parasitica
Source: Sci Rep. 2024 Mar 15;14:6307. doi: 10.1038/s41598-024-56969-4 (PMC10943224; doi:10.1038/s41598-024-56969-4)
Supplement: Supplementary file 3 — Supplementary Figure S2. [file 41598_2024_56969_MOESM3_ESM.pdf]

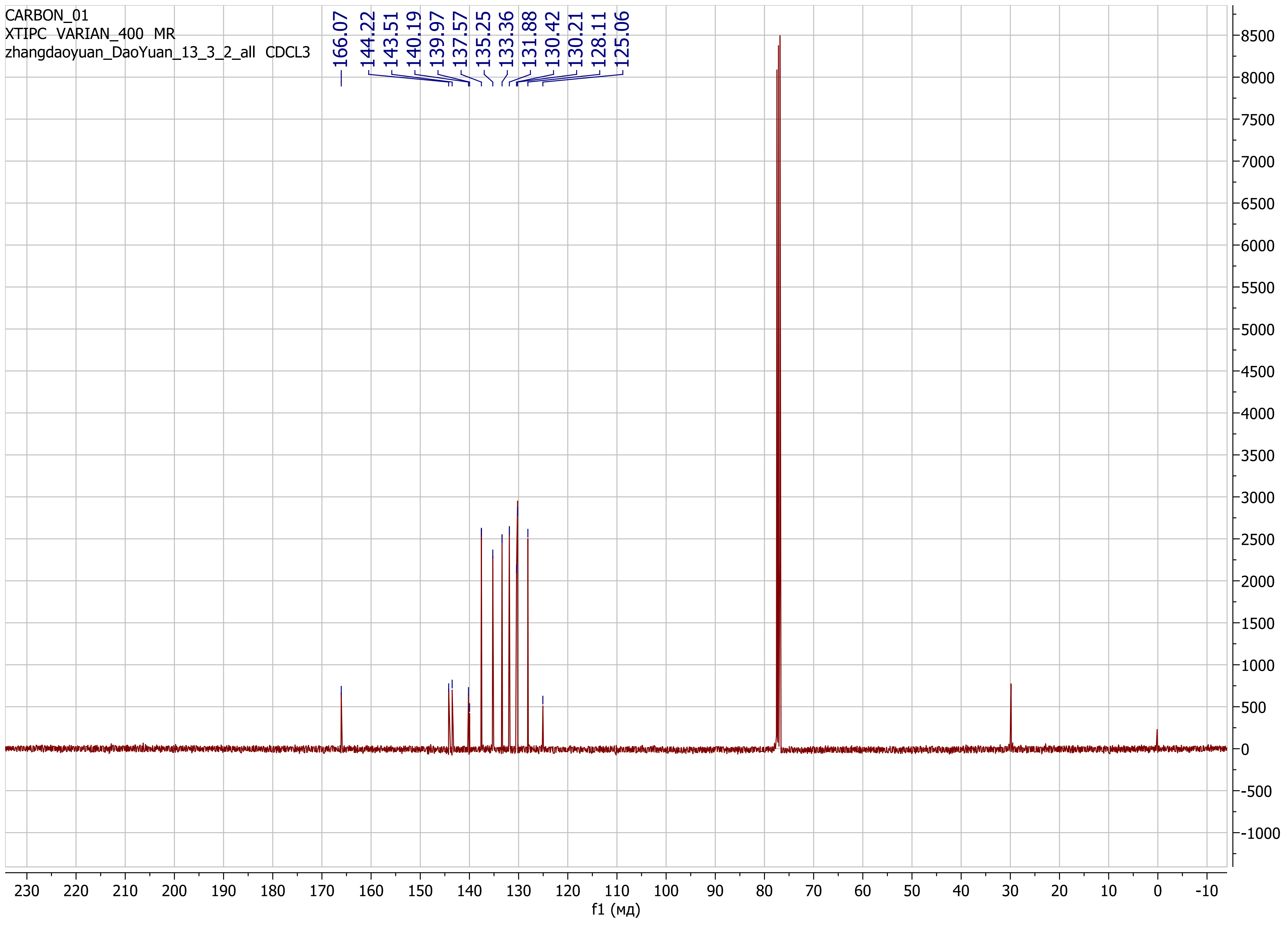
CARBON_01
XTIPC VARIAN_400 MR
zhangdaoyuan_DaoYuan_13_3_2_all CDCL3
166.07
144.22
143.51
140.19
139.97
137.57
135.25
133.36
131.88
130.42
130.21
128.11
125.06
f1 (мд)

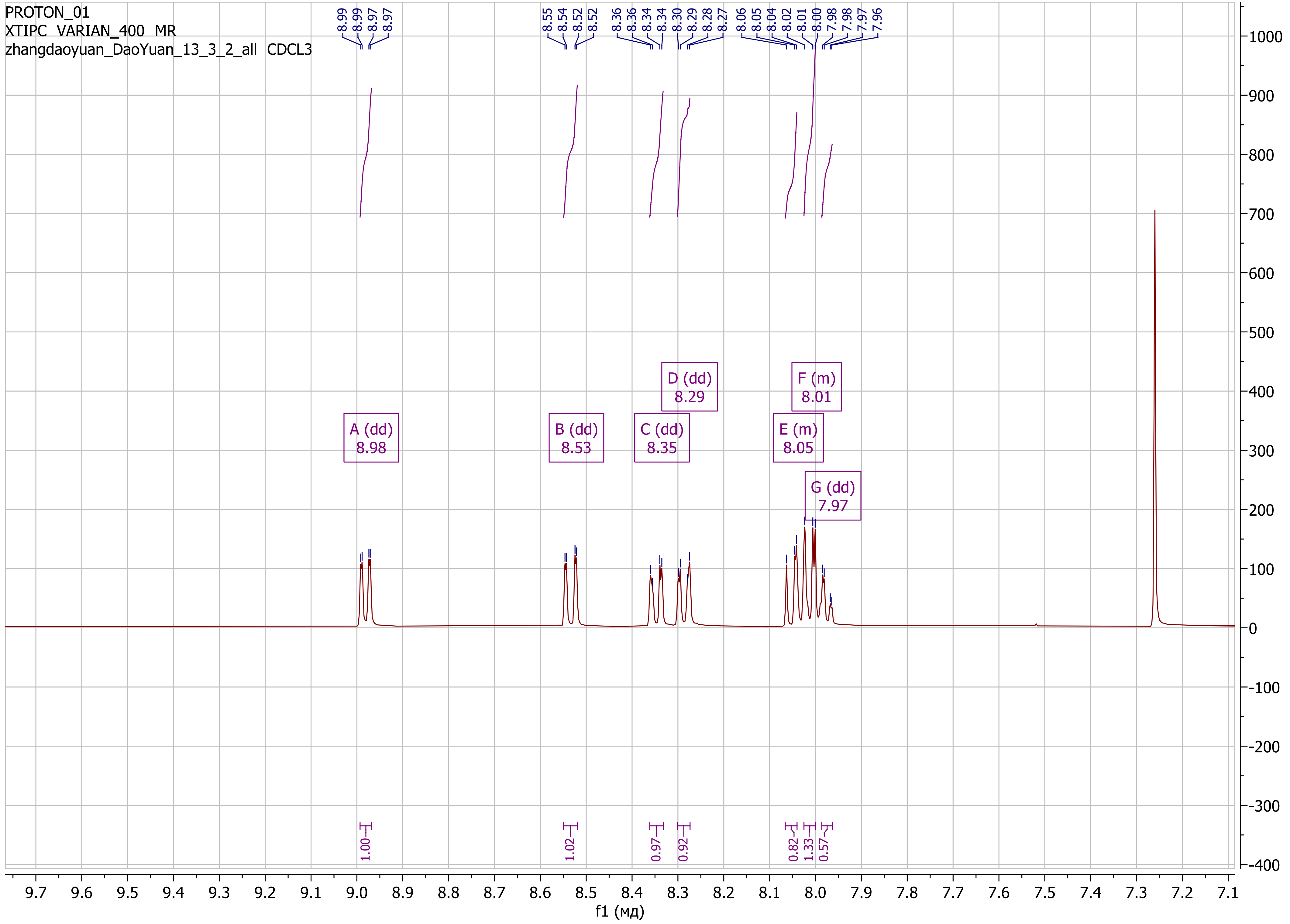
PROTON_01
XTIPC VARIAN_400 MR
zhangdaoyuan_DaoYuan_13_3_2_all CDCL3
8.99
8.99
8.97
8.97
8.55
8.54
8.52
8.52
8.36
8.36
8.34
8.34
8.30
8.29
8.28
8.27
8.06
8.05
8.04
8.02
8.01
8.00
7.98
7.98
7.97
7.96
A (dd)
8.98
B (dd)
8.53
C (dd)
8.35
D (dd)
8.29
E (m)
8.05
F (m)
8.01
G (dd)
7.97
1.00
1.02
0.97
0.92
0.82
1.33
0.57
f1 (мд)

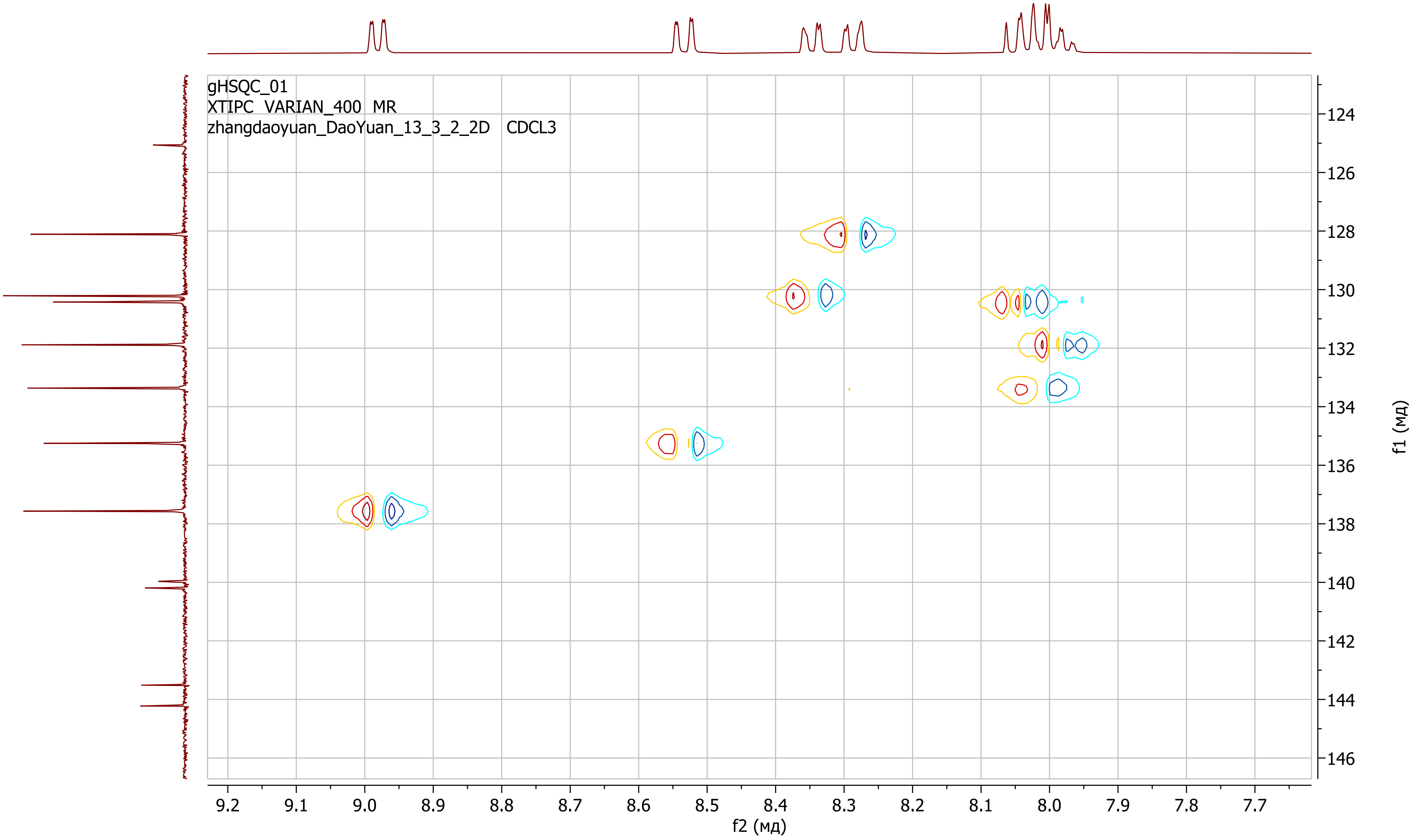
gHSQC_01
XTIPC VARIAN_400 MR
zhangdaoyuan_DaoYuan_13_3_2_2D CDCL3
f2 (мд)
f1 (мд)
9.2 9.1 9.0 8.9 8.8 8.7 8.6 8.5 8.4 8.3 8.2 8.1 8.0 7.9 7.8 7.7
124 126 128 130 132 134 136 138 140 142 144 146

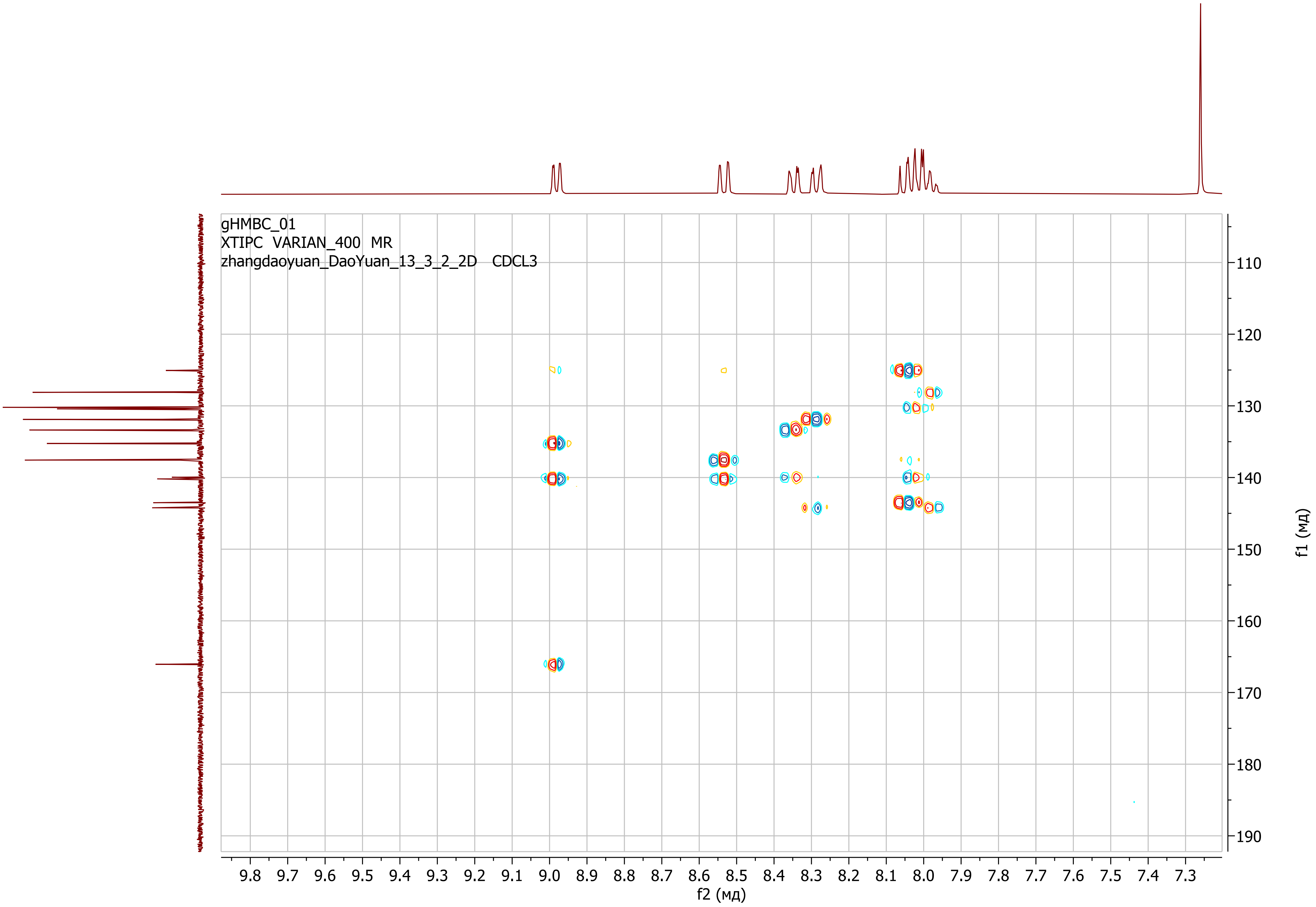
gHMBC_01
XTIPC VARIAN_400 MR
zhangdaoyuan_DaoYuan_13_3_2_2D CDCL3
f2 (мд)
f1 (мд)
9.8 9.7 9.6 9.5 9.4 9.3 9.2 9.1 9.0 8.9 8.8 8.7 8.6 8.5 8.4 8.3 8.2 8.1 8.0 7.9 7.8 7.7 7.6 7.5 7.4 7.3
110 120 130 140 150 160 170 180 190
