# Supplementary material for: Uncovering the antifungal activities of wild apple-associated bacteria against two canker-causing fungi, Cytospora mali and C. parasitica
Source: Sci Rep. 2024 Mar 15;14:6307. doi: 10.1038/s41598-024-56969-4 (PMC10943224; doi:10.1038/s41598-024-56969-4)
Supplement: Supplementary file 4 — Supplementary Legends. [file 41598_2024_56969_MOESM4_ESM.docx]

**Supplementary Data S1.** 16S gDNA sequences of 37 OTU obtained from the study.

**Supplementary Fig S1.** Mass spectra of antifungal compounds and the structure of phenazine-1-carboxylic acid (PCA).

**Supplementary Fig S2.** The raw NMR data. ^1^H and ^13^C NMR chemical shifts of phenazine-1-carboxylic acid and HMBC data in CDCL_3_, δ, ppm at 400 MHz
